# Clinical Supervisors' and Educators' Perspectives on Conditions Forming Nursing Students' Professional Identity: A Qualitative Focus Group Study

**DOI:** 10.1111/nin.70054

**Published:** 2025-09-25

**Authors:** Jette Soerensen, Mari Holen, Ida Skytte Jakobsen, Palle Larsen, Dorthe Susanne Nielsen

**Affiliations:** ^1^ UCL University College Odense Denmark; ^2^ Department of Clinical Research University of Southern Denmark Odense Denmark; ^3^ Department of Geriatric Medicine Odense University Hospital Odense Denmark; ^4^ Department of People and Technology Roskilde University Roskilde Denmark; ^5^ Department of Applied Welfare Research UCL University College Odense Denmark; ^6^ Department of Public Health Aarhus University Aarhus Denmark

**Keywords:** critical psychology, educational challenges, educators and supervisors in nursing, focus groups, nursing education, practice research, professional identity, qualitative research

## Abstract

Professional identity formation is a pivotal element of nursing education shaped by educational structures and by the roles of clinical supervisors and educators. This qualitative study examines how these stakeholders perceive their role in supporting nursing students' professional identity development and the conditions that influence this process. The empirical material was generated from six focus groups with clinical supervisors from somatic, psychiatric, and home care settings, and educators from diverse teams at a university college. Drawing on critical psychological practice research, the analysis centers on participants' interpretations, their reasons for acting as they do, and the broader societal and institutional conditions framing their practice. Three themes emerged: challenges arising from dual roles, perceived student vulnerability and its impact on the learning process, and a constructed distinction between professionalism and personality in identity formation. The findings illustrate the complex dynamics that, according to participants, shape nursing students' professional identity and highlight the importance of moving beyond explanations of individual shortcomings toward an understanding of challenges as embedded in everyday educational practices. This perspective emphasizes the need for structural changes that foster collaborative and reflective practices and better support students, clinical supervisors, and educators in navigating the complexities of nursing education.

## 1 Introduction

The formation of a professional identity among nursing students is a complex process influenced by multiple factors and conditions within both the clinical learning environment and the broader educational context. During their nursing education, students are encouraged to integrate clinical competencies with personal values, professional norms, and emotional insight, with the goal of supporting the development of a balanced and reflective professional identity. In the current study, we investigate how institutional and structural conditions influence this developmental trajectory, as understood from the perspectives of clinical supervisors and educators. By analyzing how these professionals interpret and act upon the conditions in relation to students, we aim to improve the understanding of the dynamics of support and mentorship in nursing education.

Through this research, we seek to move away from assessing actions as right or wrong toward fostering collaborative reflection and curiosity about the diverse and situated conditions that shape the educational pathways of future nursing professionals.

## 2 Background

The formation of professional identity is a crucial and broad concept in nursing education that involves the development of values, beliefs, and practices that define what it means to be a nurse (Fitzgerald 2020). This identity is shaped through everyday care experiences, formal education, clinical practice, and socialization into the profession. According to Heggen (Heggen 2019; Heggen and Terum 2017), professional identity is formed through the interaction of individual agency and social expectations, and nursing students must navigate their personal histories and experiences while aligning with the institutional arrangement of their learning path (Trede et al. 2012). Integration into a professional community of practice plays an important part in this process (Lave and Wenger 1991), and the input from clinical supervisors and educators can significantly influence the students' learning trajectories (Warne et al. 2010).

Clinical supervisors and educators assist students in connecting the theoretical knowledge and simulated experiences obtained during formal teaching to the realities of professional practice in clinical learning activities (Gillespie and McFetridge 2006; Rodrigues and Mogarro 2019). Their guidance contributes to the students understanding of caring and fosters reflection on professional practice (Fitzgerald and Clukey 2022; Sandvik and Hilli 2023). Clinical placements are designed to expose nursing students to authentic clinical settings where they can develop practical skills, apply theoretical insights, and become socially integrated into professional culture. This process involves both situated preceptorship, which occurs during daily practice, and meta preceptorship, which refers to supervisors' reflective and strategic guidance across longer learning processes that extend beyond immediate tasks (Holen and Lehn 2023). Such placements involve critical thinking, clinical decision‐making, and delivery of a high standard of patient care (Patterson et al. 2017).

Shared responsibility and social support are essential elements in helping students to manage the emotional complexity of clinical work (Soerensen et al. 2024; Vabo et al. 2022). However, inclusive education remains a challenge, especially for students with disabilities or chronic illnesses. Such students often encounter barriers that can affect their ability to participate fully in clinical education and develop a professional identity. These barriers are not only structural but also shaped by prior experiences, implicit norms, and the expectations of supervisors and educators (Moriña 2017).

Mayer et al. (2024) highlight how disability is sometimes perceived by educators and supervisors as a limitation or a risk to patient safety. These perceptions do not stem from any inadequacy on the part of the students themselves, but rather from inflexible educational structures and normative assumptions embedded in clinical and academic settings. In this sense, disability becomes constructed as a challenge within a system that often lacks the flexibility to accommodate diverse needs. Such inflexible frameworks can limit the opportunities for students to develop a sense of belonging and professional agency.

The way in which clinical supervisors and educators interpret their students abilities and potential is shaped by their own pre‐existing beliefs and professional experiences. Their understandings of what constitutes a “professional nurse” are often rooted in embodied norms and expectations that may unintentionally exclude students who do not fit the traditional mold. Clinical supervisors and educators are influenced by these preconceptions when they support or evaluate students and assess the students' emerging professional identities (Elting et al. 2021; Prude et al. 2021). Similar concerns have been raised in recent North American literature, which highlights how institutional structures, normative expectations, and limited accommodations can restrict students' opportunities to participate and form a professional identity in nursing education (Cotts 2025). In line with this, Spaan et al. (2024) argue that the integration of a disability lens is a prerequisite for inclusive higher education, as it redirects attention from individual impairments to structural and relational dimensions.

The ability of clinical supervisors and educators to fulfill their roles and promote work readiness can be influenced by social practices in nursing education and learning environments (Iskov et al. 2023). Their ability to provide meaningful learning processes can be limited by structural challenges such as high workloads, limited resources, and fragmented organizational arrangements across hospitals, home care, and educational institutions (Nielsen et al. 2017; Soerensen et al. 2024). Such conditions can restrict the opportunities for in‐depth reflection and relational engagement, both of which are essential for supporting students' professional development and identity formation (Lave and Wenger 1991).

While the importance of context is widely acknowledged in the existing literature, the focus, often on individual performance or personal limitations (even in studies among students with disabilities). This can mask the complex interplay between the structural conditions, normative assumptions, and professional practices that form students' opportunities to develop a professional identity. In the current study, we address this gap by exploring how clinical supervisors and educators perceive their roles and the conditions they work in, with the aim of contributing to a broader understanding of professional identity formation in nursing education.

## 3 THE Study

The goal of the current study is to shed light on the complex processes and challenges involved in developing nursing students' professional identities, as seen from the perspective of those directly involved in their education and training. Thus, our aim is to critically explore how clinical supervisors and educators perceive the conditions and contexts within nursing education that influence nursing students' opportunities to develop and form a professional identity.

A further aim is to understand how clinical supervisors and educators view their roles in the students' identity formation process and to identify the specific conditions they believe impact this learning trajectory.

### 3.1 Research Questions

- How do conditions and contexts influence nursing students' opportunities to develop a professional identity from the perspective of clinical supervisors and educators?
- How do clinical supervisors/educators perceive their role in the students' formation process to become nurses, and what conditions might impact this learning process?

## 4 Methods

### 4.1 Design

We designed a qualitative focus group study employing a critical psychological approach to explore the first‐person perspectives of clinical supervisors and educators regarding their role in supporting nursing students' professional identity formation.

### 4.2 Theoretical Framework

For the theoretical framework, we drew inspiration from critical psychology, a research tradition with roots in dialectic cultural‐historical psychology. Critical psychology challenges mainstream psychological theories by emphasizing the significance of social, cultural, and institutional contexts in shaping human experience, agency, and identity formation (Mørch and Huniche 2006).

Using this theoretical approach, we focus on understanding how individuals' possibilities for action and development are influenced by the broader societal structures and power relations in which they are embedded (Dreier 2007). This emphasizes the interplay between individual agency and structural forces, viewing individuals not as passive recipients of socialization but as active participants who influence and are influenced by historical and social contexts (Motzkau and Schraube 2015).

In the context of nursing education, the application of this framework allows a deeper understanding of how clinical supervisors and educators not only facilitate learning but also participate in and are influenced by the power dynamics that shape education (Mørch and Huniche 2006). We also seek to identify potential inequities and challenges within the educational system that may impact the professional development of nursing students (Dreier 2007).

By employing this critical lens, we aim to uncover how clinical supervisors and educators understand and navigate the conditions and contexts affecting nursing students' professional identity formation. We hope to gain insights into the complex relationships between individual actions and the institutional and social frameworks that shape these actions, with particular attention on the opportunities and challenges identified by clinical supervisors and educators (Motzkau and Schraube 2015).

### 4.3 Practice Research Methodology

This study is informed by practice research, a scientific methodology from the subject's standpoint that emphasizes the co‐construction of knowledge through close collaboration between researchers and practitioners (Nissen 2000). Practice research is grounded in the idea that research should generate theoretical insights about the studied phenomenon and contribute to improving practices situated in real‐world settings (Højholt and Kousholt 2019).

In the context of the current study, practice research involves the establishment of a collaborative co‐researcher group comprising nursing students, clinical supervisors, and educators. This group has met regularly throughout the study to discuss preliminary findings, reflect on analytical themes, and contribute to the development of knowledge. However, the formal data collection occurred through focus group interviews conducted with clinical supervisors and educators.

This approach seeks to democratize the research process and ensure that the voices of practitioners are central to the inquiry (Nissen 2000). Practice research also emphasizes the iterative nature of learning and change, which is particularly relevant when exploring how clinical supervisors and educators perceive and influence nursing students' identity formation. By focusing on the lived experiences and practical challenges these participants face, we seek to generate insights and practically relevant findings that can foster improvements in education and practice (Højholt and Kousholt 2019).

### 4.4 Study Setting and Recruitment

This study is the third phase of a broader Danish research study exploring how various educational and everyday life conditions influence the formation of professional identities (Soerensen et al. 2024). In the current study, we aimed to collect data from 15 educators at one university college offering a nursing baccalaureate program and 15 clinical supervisors from hospital, psychiatric, and municipal settings/primary healthcare sectors working in different clinical departments. We use purposive sampling (Malterud et al. 2016; Suri 2011) to ensure a range of perspectives. The participants were clinical supervisors and educators with a nursing background and at least six months' experience in teaching or supervising nursing students.

The director of nursing education and three clinical education coordinators were gatekeepers to the field of interest.

### 4.5 Data Collection

The empirical material was collected through six focus groups, each comprising three to six participants, during November and December 2023. Sessions were held in meeting rooms at hospitals, municipalities, or educational institutions. Focus group discussions allow dialogic interaction between participants and encourage participants to handle complex and challenging phenomena (Halkier 2010, 2020; Morgan 2010). This aligns with our practice research approach to understand individuals as interactive participants in social practices and to recognize reciprocity in dialogue. This reciprocity is crucial for how people understand their world conditions, how they articulate these conditions, and how they justify their positions, experiences, and choices (Huniche and Christensen 2023).

A focus group discussion guide was developed to ensure coverage of critical topics (Supporting Information S1). The guide was used by the researchers, primarily the first author, to structure the conversations and ensure that critical themes were addressed. It was not distributed to participants beforehand but served as a flexible guide to support the facilitation of dialogic discussions. Visual aids such as quotes from nursing students and topic keywords were prepared to enrich the discussions. For example, one quote read: *I often feel like I had to prove that I belong in the clinic*, which was used to prompt reflection on inclusion and belonging in clinical supervision. Some questions were targeted at clinical supervisors and others at educators as not all dimensions were relevant for all participants. For example, clinical supervisors were asked about their reflections on supervising and creating a safe space for learning nursing skills in the healthcare setting, while educators were asked about the content in teaching. The guide was developed based on qualitative literature and previous studies as well as discussion with the co‐researcher group. All the co‐authors and the co‐researcher group discussed and revised the guide before the focus groups were conducted.

We started each focus group by introducing all participants, explaining the research purposes, completing consent forms, and obtaining approval to record the session. We then explained confidentiality aspects, the issues to be discussed, and the group discussion process. Next, participants were asked individually to write down on Post‐it notes what they experienced to be most important in their role as supervisors or educators. After five minutes, they were asked to share their lived experiences with teaching or supervision to encourage engagement in the session. Afterward, an open question was asked where participants were encouraged to raise topics and viewpoints, they found important within the framework of the overarching phenomenon. All participants were active and shared their experiences and perspectives about their roles and attitudes toward nursing students and the nursing profession.

The first author conducted all the focus groups and led the dialogic discussions. In four of the sessions, the last author participated as a moderator, supporting the process by taking notes and ensuring that all participants were heard. In the remaining two sessions, the moderator role was filled by a nurse and a physician, both of whom were PhD students with experience in qualitative research. The interviews lasted 60 to 75 minutes and were characterized by dialogic conversation. The interviews were digitally recorded, and the first author transcribed them verbatim and validated the transcripts by listening to and reading the empirical material (Supporting Information S1: Interview Guide).

### 4.6 Data Analysis

The empirical material was analyzed using thematic analysis inspired by Braun and Clarke's reflexive approach (Braun and Clarke 2006, 2022), in alignment with our critical psychology and practice research framework (Skovhus and Thomsen 2022). This approach emphasizes the active role of the researcher in identifying patterns of meaning across the data set and acknowledges that themes are not simply discovered but constructed through interpretive engagement with data.

The analysis was an iterative and collaborative process. The first author transcribed all focus group interviews verbatim and immersed herself in the data by reading and re‐reading the transcripts. Initial notes and reflections were documented in analytic memos. In the second phase, preliminary codes were generated inductively, focusing on participants' experiences, dilemmas, and meaning‐making related to their roles as clinical supervisors and educators.

These codes were then discussed in the co‐researcher group, which included nursing students, clinical supervisors, and educators. Their insights helped refine the coding framework and ensured that the analysis remained grounded in practice. In the third phase, the research team (the co‐authors) collaboratively reviewed and grouped the codes into broader themes, guided by the research questions and theoretical framework. This process involved multiple rounds of discussion and refinement to ensure coherence and depth.

We applied a “conditions, meanings, and reasons” approach to our analysis, by examining the interconnection between objective conditions, their interpretations, and the reasons behind actions. Societal factors shape conditions, and our focus was on exploring how participants respond to their perceived possibilities and limitations for action, and how these responses, in turn, reshape those conditions (Skovhus and Thomsen 2022).

Personal meanings refer to how individuals interpret the conditions they encounter. In this analysis, we explored the interpretations that clinical supervisors and educators assign to these conditions, recognizing that these interpretations influence the actions they perceive as available to them and the challenges they face (Bøttcher et al. 2018). Subjective reasons are the intentions and motivations underlying individuals' actions (Bøttcher et al. 2018).

Throughout the analysis, we engaged in reflexive dialogue about our own positions, assumptions, and interpretations. We paid particular attention to how power relations, institutional structures, and professional norms shaped participants' narratives.

The analysis process described above is illustrated in Supporting information S2. Illustration of the analysis process resulting in Theme 2, “Perceived student vulnerability and its impact on the learning process”

### 4.7 Transparency and Trustworthiness

To accurately report important perspectives and nuances, the findings were discussed and reviewed by all members of the author group (Ahmed 2024).

### 4.8 Ethical Considerations

The study was conducted in compliance with the Declaration of Helsinki (World Medical Association 2025) and the guidelines set by the Regional Committees on Health Research Ethics (Project ID: 20222000‐37). According to Danish law, the study did not require formal ethical approval.

All focus group members consented to participate after being given written and verbal information about the study. All issues related to ethical considerations were considered throughout the study to protect the participants' privacy, confidentiality, and comfort. Written and oral informed consent was gained from the participants at the start of the study. Participants were assured that they could withdraw from the study at any time. All records and study transcripts were stored in a locked filing cabinet, and all electronic data were password‐protected.

### 4.9 Rigor and Reflexivity

As a researcher (PhD student) in this study, the first author actively engaged in reflexivity to acknowledge how her background as a nurse and lecturer might influence the interpretations and interactions with participants. In line with critical psychology, she examined her role as a co‐participant in the social practices of nursing education (Dreier 1999; Højholt 2022). During the group discussion, she aimed to create an environment for shared exploration and inquiry and used a reflexive log throughout the study to document her reflections. Reflexivity was a shared commitment across the research team. All co‐authors engaged in ongoing discussions about how their professional backgrounds, assumptions, and institutional affiliations shaped the research process and interpretation of data. All members of the research team are experienced lecturers in nursing education and are experienced in qualitative research. Our use of collaborative reflexivity was in line with the approach of Schraube and Højholt's (2016) and allowed us to deepen our curiosity and become shared co‐producers of interpretations and knowledge. Dreier (2007) and Højholt (2022) underscore the importance of seeing researchers as situated in the practices they study, with a responsibility to acknowledge how institutional contexts impact both participants and researchers. By remaining reflexive, the first author and the research team (co‐authors) aimed to bring transparency and trustworthiness to the research processes.

## 5 Findings

### 5.1 Characteristics of Participants

As two clinical supervisors were unable to participate in the study (one due to illness and the other a scheduling conflict), the focus groups included 28 participants. Participants ranged in age from 26 to 74 years.

Of the 13 clinical supervisors, five worked in hospitals, three in psychiatric departments, and five in nursing home care. Each clinical supervisor held a diploma in guidance, and three had additional academic qualifications. Their nursing experiences ranged from 2 to 37 years across various medical specialties, and their experiences in preceptorship varied from 1 to 37 years. The 15 educators were employed at a major nursing school and taught students in the first to seventh semesters. All educators held master's degrees, with one also holding a doctoral degree, and two were PhD students. Their teaching experience ranged from 8 months to 21 years.

Participants are identified in this paper by ID numbers [1–28]. When presenting quotes, we indicate the speaker's role (e.g., clinical supervisor or educator) to provide context and clarity.

### 5.2 Findings

**Figure 1:**
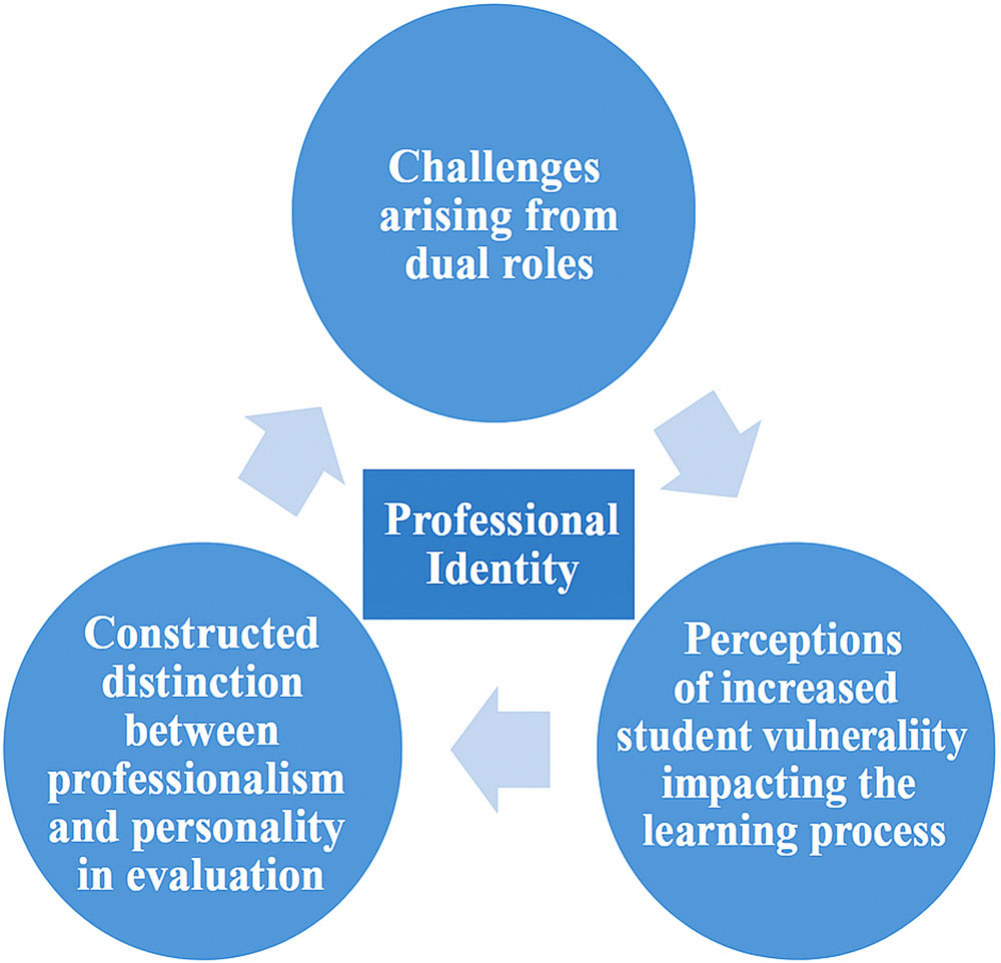
Illustration of the interconnected themes.

Three interconnected themes emerged from the focus group discussion (see Figure 1). These are presented here in turn: challenges arising from dual roles, perceived student vulnerability and its impact on the learning process, and constructed distinction between professionalism and personality.

### 5.3 Challenges Arising From Dual Roles

The discussion in the focus groups revealed several contextual and structural conditions that influenced the experiences of clinical supervisors and educators. Participants described the challenges of navigating dual roles that, were influenced by organizational frameworks and their own interpretations of professional responsibilities. For clinical supervisors, this involved balancing the demands of guiding and mentoring nursing students while simultaneously ensuring high‐quality patient care. Educators, in turn, were responsible for teaching and academic mentoring, often alongside administrative or institutional obligations.

Several clinical supervisors referred to their roles as “two jobs in one,” highlighting how clinical responsibilities often competed for time and attention with student supervision. This tension in everyday social practice limited their ability to act as role models, something they viewed as essential for students' professional identity formation. Some clinical supervisors described structural constraints such as insufficient time for student supervision, highlighting a power imbalance between institutional demands and the agency required for supervision. One clinical supervisor reflected on this experience:

We had two jobs in one, but not equal time for each. We had to find a little time for the supervisory role in between.

(ID16)

These structural constraints influenced how some clinical supervisors perceived their roles, emphasizing their growing sense that educational responsibilities were increasingly challenging and undervalued compared to clinical tasks. The meanings derived from these conditions indicated that time limitations not only restricted their ability to support students but also affected their self‐understanding as role models. Clinical supervisors noted that the allocated five hours per week per student were insufficient. These five hours included not only face‐to‐face supervision but also time spent on planning, preparation, documentation, and organizing the clinical learning process. Supervisors expressed that this limited timeframe constrained their ability to provide meaningful pedagogical support and individualized guidance. The explanations clinical supervisors gave for their actions reflected the necessary prioritization of clinical work over student supervision. They often experienced conflicts between their clinical work and their educational commitments. One clinical supervisor illustrated these dilemmas with an example:

I could relate to the need to fight for the five hours a week we were supposed to have. Supervising wasn't our main task—our primary role was as nurses, and citizens always come first. That's how it should be, but we still needed time to evaluate our progress and plan supervision. It takes a lot of time and required good planning, knowing everything could fall apart if someone called in sick or patients needed urgent care. Still, that time was important.

(ID18)

Most clinical supervisors emphasized that their priorities were largely determined by the conditions of the work environment, where patient care was viewed as the most immediate and pressing responsibility. Despite this, they recognized their roles within these communities of practice (context) that shaped students' opportunities to understand the complexity of nursing. Thus, clinical supervisors believed it was essential for them to spend time with students during their learning processes.

Educators described their dual responsibilities as organizing learning activities and building supportive relationships with students. They wanted the students to acquire theoretical knowledge, develop critical thinking, and understand the professional standards necessary to become nurses. However, they also shared that their self‐understanding as educators was influenced by time pressures, administrative tasks, and standardized teaching requirements. This balance between organizational conditions and individual interpretation was important for understanding the challenges they faced in their roles. An educator shared her experiences:

It was difficult to differentiate teaching. I didn't know students' starting points, and the framework wasn't designed for that. It ended up being about catering to the lowest common denominator.

(ID4)

These accounts illustrate how both clinical supervisors and educators navigated tensions between institutional expectations and their own professional values. Their experiences underscore how dual‐role challenges are not only individual struggles but are shaped by broader organizational structures and conditions.

### 5.4 Perceived Student Vulnerability and Its Impact on the Learning Process

Clinical supervisors and educators identified several challenges and opportunities when supervising and teaching students. They described the increasing diversity among students as a significant factor influencing the existing framework and their perceived ability to support students' development of nursing competencies. While previous student cohorts were described as more homogeneous, participants noted a shift toward greater diversity in students' backgrounds and needs.

This shift was perceived as introducing new challenges, including what participants referred to as “increased vulnerability,” “more diagnoses,” and “mental health challenges.” These terms reflected the complexity they observed in the student population and how this complexity disrupted established routines and expectations in supervision and teaching. These perceptions were shaped not only by individual student characteristics but also by structural conditions such as time constraints and rigid educational arrangements, which influenced how supervisors and educators understood their roles and agency. One clinical supervisor remarked:

I think our framework was challenged. Also, because the students demanded more today. Unfortunately, many students come with something in their background, whereas before, it was a more homogeneous group. It was not the same, and that was what we had to deal with.

(ID16)

In this study, we use the term *perceived vulnerability* to reflect how supervisors and educators constructed their experiences with students, and to avoid labeling the students as vulnerable. This framing allowed us to explore how vulnerability is socially and institutionally produced, and how it shaped pedagogical relationships and expectations in clinical education.

The analysis also highlighted how structural conditions limited students' participation, often amplifying attention to individual vulnerabilities rather than addressing systemic inequities. For instance, one clinical supervisor noted how students often introduced themselves by stating their diagnoses, which they felt came to define the students' identities: “*It was striking that this is how they present themselves […] they forgot who they were besides this. The focal point of the guidance became the student's vulnerability, which easily took the focus away from professional learning in practice*” [ID22]. This pattern reflects how broader societal discourses around mental health and responsibility shaped how students engaged in learning environments.

Many educators and clinical supervisors emphasized the importance of having time to establish trusting and supportive relationships with students, particularly those who needed help structuring everyday life, including study routines. Although they attempted to foster connections by scheduling informal conversations, these efforts were often perceived as “extra” rather than integral to their core responsibilities.

Time and structure were key barriers. As a teacher, I often rushed in and out […], leaving little time to build trust with students. Sometimes, I couldn't even respond to emails, which fostered mistrust […] The lack of time makes it difficult to connect, whether through conversations, emails, or hallway encounters.

(ID11)

Participants described their roles as both academic and relational, and they viewed trust‐building as essential to supporting students' professional identity formation. These relational dynamics also influenced educators' own professional identity, sense of agency, and ability to serve as role models.

Another challenge was the tension between respecting students' privacy (under GDPR) and ensuring adequate support for both students and patients. The limited information that supervisors and internship sites received about students' disabilities reduced the ability to ensure patient safety and provide appropriate support. Some supervisors expressed relief at not having prior knowledge about a student's background as it allowed them to approach students without a preconceived understanding. However, others noted that the absence of information could lead to students being placed in roles that exceeded their agency.

Due to GDPR, we received almost no information […] We needed to ensure that our patients received respectful treatment, so it was important to know certain information to support both the student and protect the patient.

(ID18)

Clinical supervisors and educators expressed concern that the students vulnerabilities the perceived could hinder both engagement in learning and the formation of a professional identity. Some questioned whether students facing significant challenges should reconsider their career path, given the demands of the nursing profession. Others reflected on the lack of flexibility in nursing education compared to workplace accommodations. An educator remarked:

In any case, I think there is an inequity in that you had to be a full‐time student. If you had a chronic illness or something else, then afterward, you could get something that corrects it. Why couldn't we also have those conditions during the educational path? That surprises me.

(ID4)

This highlighted how educators perceived the structural constraints that affect students' ability to become nurses and how institutional frameworks influenced their agency to provide support. These conditions hindered the students' ability to maintain cohesion in their lives and pursue their professional goals, inadvertently reinforcing inequity and limiting meaningful engagement.

### 5.5 Constructed Distinction Between Professionalism and Personality

The focus groups revealed that the institutionalized feedback culture in nursing education often posed challenges for clinical supervisors and educators in providing feedback within a holistic framework. Standardized feedback systems emphasized technical and academic performance but frequently overlooked the relational aspects essential for shaping students' professional identity. This discrepancy created tension between adhering to formal feedback structures and addressing the more nuanced, formative needs of students. Clinical supervisors and educators balanced these competing demands by combining structured assessment with the cultivation of trust, empathy, and humanity in the learning process.

They explained that the profession often focuses on a narrow ideal of what constituted a “good nurse” and discussed the evaluation of whether a student met these standards. The power of institutionalized normative expectations could influence the development of learning environments and pressured some students to conform, which limited their agency and learning path in nursing.

One educator noted a tendency within the profession to promote a narrow and stereotypical image of what it means to be a “real nurse.”

This highlighted the tension between individuality and conformity within the profession. While some clinical supervisors and educators valued unique expressions of professional identity, students were often influenced by the implicit norms of being a “good nurse” and “good student.”

It's a balancing act […] we had ethical guidelines and codes of professionalism that we must work within, but we are also different people. There may have been a tendency to believe there was only one way to be a good nurse and student. It could feel narrow, stereotypical, and culturally determined.

(ID9)

Educators discussed their understanding of nursing and professional identity, noting that they sometimes found it challenging to articulate the essence of nursing. However, they believed that reflections on practice helped students strengthen their sense of self. While relational skills were crucial in nursing, all clinical supervisors and educators acknowledged that opportunities for feedback on these aspects were limited.

Clinical supervisors and educators noted that the dominant framework for assessment was academic performance, with little value placed on the relational and practical competencies essential to nursing. They reflected on the difficulty of recognizing and providing feedback on these competencies, which are often abstract and intangible. One educator explained:

We were also talking about certain qualities that were much harder to measure, harder to give feedback on. It must be incredibly difficult for students themselves to assess whether they were good enough because it was so abstract […] Maybe we needed to pay more attention to recognizing some of the competencies they have that they struggled to see.

(ID10)

Some supervisors addressed these gaps informally by providing feedback through personal notes at the end of internships, despite such practices not being formally recognized. One supervisor shared:

I gave them a little note that they could hang on their fridge. It might not be long, just a few words about what the student was good at. I hoped it gave them a positive push to become proud, good nurses.

(ID17)

Clinical supervisors and educators described how some students struggled to understand study norms, which could increase their feelings of vulnerability if they felt different or “wrong” in that environment. Supervisors and educators faced a dilemma as to whether they should address aspects of a student's personality when these conflicted with the profession's standards. As one educator remarked:

One of the hardest things was when supervisors said that the student could do everything but seemed indifferent and lacked empathy toward patients. It was difficult because it was about the student's personality, not the professional side […] it became a gray area.

(ID9)

One educator reflected on how fear of overstepping professional boundaries might lead them to avoid personal engagement with students, resulting in a narrow focus on professionalism.

The analysis revealed that clinical supervisors and educators distinguished between professional skills and personal qualities. While formal feedback focused on technical skills, the reflections of supervisors and educators highlighted an underlying tension regarding how to engage with students' traits. Implicit norms shaped the boundaries of acceptable behavior in clinical practice and classroom teaching. Although these norms were not always explicitly stated, they could influence students' experiences, impacting how they internalized professional values and developed their professional identities.

## 6 Discussion

The findings of the current study contribute to a more nuanced understanding of the challenges and contextual conditions encountered by clinical supervisors and educators in their efforts to support nursing students' professional identity formation. The application of critical psychology as an analytical framework facilitated an in‐depth exploration of the dynamic interplay between objective conditions, subjective meanings, and the reasons that guide professional actions. By focusing on how clinical supervisors and educators interpret their roles, we have identified tension related to the dual responsibilities of clinical supervisors and educators, limitations in learning practices, and challenges associated with professional norms.

In relation to the theme of dual‐role challenges, clinical supervisors and educators reported having to navigate competing obligations shaped by their differing responsibilities toward students and the broader social practice of clinical work and nursing education. These competing demands often limit their ability to fulfill their educational obligations to students and contribute to a perception that their educational responsibilities are undervalued. This reflects a broader conflict between workplace identity and role, where power imbalances and contradictory expectations can undermine an individual's self‐understanding and professional identity. Heggen (2019) emphasizes that professional identity is a dynamic and ongoing process shaped by individuals' experiences of autonomy and recognition in their work. From a critical psychological perspective, the concept of *everyday conflictuality* (Schraube and Jartoft 2017) highlights how internal and external tension conflicts are not merely barriers but can serve as productive forces for critical reflection and transformation in situated practice. For instance, conflict between patient care and student supervision, or between teaching and administrative duties, may generate frustration but also encourage critical engagement with professional roles.

The growing diversity among nursing students, including variations in life experiences and backgrounds, is challenge to traditional supervisory and pedagogical approaches. From a critical psychology standpoint, vulnerability is not viewed as an inherent personal trait but as something that emerges through interaction with specific structural and relational conditions. This perspective is supported by Vestphal et al. (Vestphal et al. 2020), who argue that students' diverse experiences bring valuable insights but also require more flexible and inclusive educational practices. Research on inclusive learning environments in nursing (Charania and Patel 2022; Cotts 2025; Metzger and Taggart 2020) further suggests that addressing these diverse needs involves rethinking institutional priorities and pedagogical strategies to better support students in managing the intersection of personal, academic, and professional demands.

Clinical supervisors carry an ethical responsibility to ensure patient safety and uphold standards of care. In some cases, this responsibility leads to the view that students should be guided out of the nursing profession if they are unable to meet the essential competencies. This adds an extra layer of complexity to the supervisory role, as supervisors must balance the needs and potential of diverse students with the imperative to maintain high standards in clinical practice.

Rather than attributing challenges in participation to individual students' psychological traits, lack of readiness, or generational characteristics, the focus should shift toward adapting institutional and practice‐based frameworks to promote equitable participation. The historical tendency has been to individualize conflicts, but the dialectic approach of critical psychology (Dreier 2009) emphasizes the interconnection between personal and contextual factors, as well as between knowledge and action in practice. This tendency to individualize is reinforced by dominant discourses of “normalcy” in higher education, which construct a binary between “traditional” and “nontraditional” students (Gibson et al. 2016). This framing positions diverse students as “other” and places the burden of adaptation on them, rather than prompting institutions to critically examine and adjust existing educational structures.

The institutionally constructed distinction between professionalism and personality in evaluation practices reveals a complex relationship between the personal and professional domains in nursing education. This distinction is shaped by standardized assessment systems, institutional norms, and neoliberal influence on education (Gibbons 2018). Clinical supervisors and educators often struggle to reconcile formal academic assessments with the need to support relational competencies and personal development, both of which are central to professional identity formation. This constructed distinction reflects concerns raised by Collier‐Sewell and Monteux (2024), who argue that the use of overly standardized approaches run the risk of narrowing the scope of what is recognized as professional identity.

Implicit norms about what constitutes a “good nurse” constrain individual expression and promote a standardized model of professional identity. This resonates with Owens et al. (2024), who argue that dominant models of professionalism in North American nursing education often rely on narrow behavioral standards that may exclude students who do not conform to traditional norms. Echoing these concerns, several educators in our study expressed uncertainty about how to address students' personal characteristics when these appeared to conflict with professional expectations. This ambiguity reflects a broader cultural hesitation within nursing education to integrate personal and professional dimensions, often due to concerns about overstepping boundaries.

Norm critique is a valuable analytical lens for examining these dynamics. By questioning the implicit assumptions that define the “ideal” nurse, it becomes possible to challenge the frameworks that shape educational and clinical practices in nursing. The findings suggest that dominant ideals, such as emotional resilience and stability, may marginalize students who do not conform to these expectations. This aligns with research and disability studies showing that disabled individuals are often expected to conform to meet able‐bodied norms to be accepted into healthcare professions (Bogart and Dunn 2019; Mayer et al. 2024). From a critical psychological perspective, such expectations reinforce existing power structures and position some students as “difficult” or “deviant.”

This also relates to how students' opportunities for participation and identity formation can be limited when they are externally perceived as “vulnerable.” Spiers (2000) distinguishes between self‐experienced (emic) and externally attributed (etic) vulnerability, arguing that vulnerability is socially constructed rather than an individual trait. Being labeled as vulnerable can reinforce marginalization, a view that aligns with our findings and with critical psychological perspectives. Recognizing this shifts the focus from individual deficits to contextual conditions, supporting more inclusive approaches in nursing education.

Norm critique thus encourages a shift from viewing these students as problems to be managed, toward recognizing the limitation of current frameworks (Tengelin et al. 2020), the need for more inclusive conditions for professional identity formation (Lewis et al. 2021), and the advantages of a different approach to evaluating and determining professionalism (Mayer et al. 2024).

### 6.1 Study Strengths and Limitations

By collecting insights from both clinical supervisors and educators, we have achieved a more holistic perspective on professional identity formation and the opportunities for strengthening cohesive support systems. We provide participants with space and time to reflect on students' learning trajectories and their own roles, thus helping to uncover implicit assumptions and practices that might otherwise have remained unexamined.

However, we might have improved the consistency of the data collection had we used the same moderator and note‐taker across all focus groups. Conducting a second focus group with each participant group might have deepened and expanded the reflections and allowed us to further clarify the emerging themes.

The demographic composition of the research team and the participants could have been a limitation. The researchers and most participants shared similar professional and cultural backgrounds, which may have influenced the interpretation of data, particularly in relation to understanding the diversity of student experiences and identity formation. This relative homogeneity may have limited our analytical sensitivity to nuances related to ethnicity, gender identity, disability, or other dimensions of difference that shape students' trajectories in nursing education. From a critical psychological perspective, such positionalities are not neutral but shape how meaning is constructed and how certain experiences are recognized or overlooked. Future research would benefit from involving more demographically diverse research teams and participant groups to better capture the complexity of student diversity and its implications for professional identity formation.

### 6.2 Recommendations for Further Research

As nursing student populations become increasingly diverse, further research is warranted to explore how students from various backgrounds experience identity formation, including the specific challenges and opportunities they encounter. Future studies could examine how educational arrangements and pedagogical approaches can be designed to promote inclusive participation and ensure equitable support for all students in developing their professional identities.

Additionally, research should investigate the institutional and structural condition that shape the roles of clinical supervisors and educators in this process. An exploration of how different organizational models, resource allocations, and workload expectations can influence the capacity of clinical supervisors and educators to support their students' development could help identify best practices and potential areas for systemic change.

### 6.3 Implications for Policy and Practice

The findings of this study indicate a need for structural adjustments in nursing education policy to better support clinical supervisors and educators in managing their dual responsibilities. Institutional frameworks should be revised to address the constraints these professionals face, thereby enabling more flexible and relational forms of support that contribute to students' professional identity formations.

Furthermore, policy initiatives should actively promote diversity and inclusion by implementing participation conditions that accommodate a wide range of students' needs. Such measures are essential to prevent educational inequities and to ensure that all students have the opportunity to engage meaningfully in their professional development.

## 7 Conclusion

The findings of this study underscore the need for a critical re‐evaluation of the complex challenges faced by clinical supervisors and educators in nursing education. Opportunities for holistic and meaningful feedback are limited by current institutional practices, such as standardized feedback system. At the same time, increasing student diversity presents additional challenges for educators seeking to support individual learning and identity development.

The dual role demands of balancing mentorship with patient care or administrative responsibilities are shaped by organizational structures that often restrict flexibility and adaptability. The study findings suggest that we need to develop inclusive and responsive educational frameworks to resolve these constraints. Instead of the challenges being interpreted as individual shortcomings, they should be understood within the broader context of everyday educational practices. This perspective highlights the importance of structural reforms that prioritize collaborative and reflective approaches to support both students and educators/clinical supervisors in the ongoing process of professional identity formation.

## Author Contributions

All authors meet the authorship criteria recommended by the ICMJE (https://www.icmje.org/icmje-recommendations.pdf). All authors contributed to the study conception and design. Material preparation and data analysis were performed collaboratively. Jette Soerensen drafted the initial manuscript. All authors provided critical feedback and revisions, and all approved the final version.

## Ethics Statement

In accordance with Danish legislation, qualitative studies are not subject to mandatory registration. The study was submitted to the Regional Committees on Health Research Ethics for Southern Denmark (Project ID: 20222000‐37), and all relevant permissions were obtained.

## Consent

This study adheres to the Danish Code of Conduct for Responsible Research. All participants were informed verbally and in writing about the aim and content of the study, confidentiality, participation being voluntary and their right to withdraw at any time.

## Conflicts of Interest

The authors declare no conflicts of interest.

## Supporting information

Supplementary information 1. Interview guide.

Supplementary information 2. Illustration of the analysis process.

## Data Availability

The data that support the findings of this study are available on request from the corresponding author. The data are not publicly available due to privacy or ethical restrictions.

## References

[nin70054-bib-0001] Ahmed, S. K. 2024. “The Pillars of Trustworthiness in Qualitative Research.” Journal of Medicine, Surgery, and Public Health 2: 100051. 10.1016/j.glmedi.2024.100051.

[nin70054-bib-0002] Bogart, K. R. , and D. S. Dunn . 2019. “Ableism Special Issue Introduction.” Journal of Social Issues 75, no. 3: 650–664. 10.1111/josi.12354.

[nin70054-bib-0003] Braun, V. , and V. Clarke . 2006. “Using Thematic Analysis in Psychology.” Qualitative Research in Psychology 3, no. 2: 77–101. 10.1191/1478088706qp063oa.

[nin70054-bib-0004] Braun, V. , and V. Clarke . 2022. Thematic Analysis: A Practical Guide. SAGE.

[nin70054-bib-0005] Bøttcher, L. , D. Kousholt , and D. Winther‐Lindqvist . 2018. “Indledende refleksioner over analyseprocesser og kvalitetsdimensioner. [Initial Reflections on Analysis Processes and Quality Dimensions].” In Kvalitative analyseprocesser: med eksempler fra det pædagogisk psykologiske felt, edited by L. Bøttcher , D. Kousholt , and D. Winther‐Lindqvist , 17–38. Samfundslitteratur.

[nin70054-bib-0006] Charania, N. A. M. A. , and R. Patel . 2022. “Diversity, Equity, and Inclusion in Nursing Education: Strategies and Processes to Support Inclusive Teaching.” Journal of Professional Nursing 42: 67–72. 10.1016/j.profnurs.2022.05.013. 36150880

[nin70054-bib-0007] Collier‐Sewell, F. , and S. Monteux . 2024. “What Is the Purpose of Nurse Education (And What Should It Be)?” Nursing Inquiry 31, no. 3: e12640. 10.1111/nin.12640. 38685718

[nin70054-bib-0008] Cotts, K. G. 2025. “Disability‐Inclusive Accommodations in Nursing Education—Addressing Health Equity Needs.” JAMA Network Open 8, no. 2: e2461044. 10.1001/jamanetworkopen.2024.61044. 39976972

[nin70054-bib-0009] Dreier, O. 1999. “Personal Trajectories of Participation Across Contexts of Social Practice.” Outlines. Critical Practice Studies 1, no. 1: 5–32. 10.7146/ocps.v1i1.3841.

[nin70054-bib-0010] Dreier, O. 2007. Psychotherapy in Everyday Life. Cambridge University Press. 10.1017/CBO9780511619519.011.

[nin70054-bib-0011] Dreier, O. 2009. “Persons in Structures of Social Practice.” Theory & Psychology 19, no. 2: 193–212. 10.1177/0959354309103539.

[nin70054-bib-0012] Elting, J. K. , E. Avit , and R. Gordon . 2021. “Nursing Faculty Perceptions Regarding Students With Physical Disabilities.” Nurse Educator 46, no. 4: 225–229. 10.1097/NNE.0000000000000940. 33196591

[nin70054-bib-0013] Fitzgerald, A. 2020. “Professional Identity: A Concept Analysis.” Nursing Forum 55, no. 3: 447–472. 10.1111/nuf.12450. 32249453

[nin70054-bib-0014] Fitzgerald, A. , and L. Clukey . 2022. “Factors Influencing Nursing Professional Identity Development: A Qualitative Study.” Nursing Forum 57, no. 6: 1346–1353. 10.1111/nuf.12816. 36259223

[nin70054-bib-0015] Gibbons, A. 2018. “Neoliberalism, Education Policy and the Life of the Academic: A Poetics of Pedagogical Resistance.” Policy Futures in Education 16, no. 7: 918–930. 10.1177/1478210318774675.

[nin70054-bib-0016] Gibson, S. , D. Baskerville , A. Berry , A. Black , K. Norris , and S. Symeonidou . 2016. “Diversity’ ‘Widening Participation’ and ‘Inclusion’ in Higher Education: An International Study.” Widening Participation and Lifelong Learning 18, no. 3: 7–33. 10.5456/WPLL.18.3.7.

[nin70054-bib-0017] Gillespie, M. , and B. McFetridge . 2006. “Nurse Education – The Role of the Nurse Teacher.” Journal of Clinical Nursing 15, no. 5: 639–644. 10.1111/j.1365-2702.2006.01344.x. 16629973

[nin70054-bib-0018] Halkier, B. 2010. “Focus Groups as Social Enactments: Integrating Interaction and Content in the Analysis of Focus Group Data.” Qualitative Research 10, no. 1: 71–89. 10.1177/1468794109348683.

[nin70054-bib-0019] Halkier, B. 2020. “Fokusgrupper [Focusgroup].” In Kvalitative metoder ‐ en grundbog, 3rd ed., edited by S. Brinkmann and L. Tanggaard , 167–184. Hans Reitzels Forlag.

[nin70054-bib-0020] Heggen, K. 2019. “Profesjon og identitet. [Profession and identity].” In Profesjonsstudier, 4th ed., edited by A. Molander and L. I. Terum , 321–332. Universitetsforlaget.

[nin70054-bib-0021] Heggen, K. , and L. I. Terum . 2017. “The Impact of Education on Professional Identity.” In Social and Caring Professions in European Welfare States: Policies, Services and Professional Practices, edited by B. Blom , L. Evertsson , and M. Perlinski , 21–36. Policy Press Scholarship Online. 10.1332/policypress/9781447327196.003.0002.

[nin70054-bib-0022] Holen, M. , and S. Lehn . 2023. “Meta Preceptorship and Situated Preceptorship in Nursing: A Conceptual Proposal.” Klinisk Sygepleje 37, no. 3: 193–204. 10.18261/ks.37.3.6.

[nin70054-bib-0023] Huniche, L. , and H. M. Christensen . 2023. *Praksisforskning på sundhedsområdet: en metodebog* [Practice Research in Healthcare: A Methodology Handbook]. Munksgaard.

[nin70054-bib-0024] Højholt, C. 2022. “Kvalitativ forskning om situeret ulighed i skolen [Qualitative Research on Situated Inequality in Schools].” Qualitative Studies 7, no. 1: 86–111. 10.7146/qs.v7i1.133068.

[nin70054-bib-0025] Højholt, C. , and D. Kousholt . 2019. “Developing Knowledge Through Participation and Collaboration: Research as Mutual Learning Processes.” Annual Review of Critical Psychology (Online) 16, no. 16: 575–604. https://discourseunit.com/annual-review/arcp-16-kritische-psychologie-2019/.

[nin70054-bib-0026] Iskov, T. , K. Heggen , J. G. Glavind , and V. R. Noer . 2023. “Kohærens og professionel identitet: Et begrebsapparat, som kan styrke professionsuddannelser?” Tidsskrift for Professionsstudier 19, no. 36: 6–15. 10.7146/tfp.v19i36.139968.

[nin70054-bib-0027] Lave, J. , and E. Wenger . 1991. Situated Learning: Legitimate Peripheral Participation. Cambridge University Press.

[nin70054-bib-0028] Lewis, L. , D. Biederman , D. Hatch , A. Li , K. Turner , and M. A. Molloy . 2021. “Outcomes of a Holistic Admissions Process in an Accelerated Baccalaureate Nursing Program.” Journal of Professional Nursing 37, no. 4: 714–720. 10.1016/j.profnurs.2021.05.006. 34187669

[nin70054-bib-0029] Malterud, K. , V. D. Siersma , and A. D. Guassora . 2016. “Sample Size in Qualitative Interview Studies.” Qualitative Health Research 26, no. 13: 1753–1760. 10.1177/1049732315617444. 26613970

[nin70054-bib-0030] Mayer, Y. , L. Nimmon , M. Shalev , et al. 2025. “Belonging in Dual Roles: Exploring Professional Identity Formation Among Disabled Healthcare Students and Clinicians.” Advances in Health Sciences Education: Theory and Practice 30: 1101–1121. 10.1007/s10459-024-10386-4. 39509065 PMC12390862

[nin70054-bib-0031] Metzger, M. , and J. Taggart . 2020. “A Longitudinal Mixed Methods Study Describing 4th Year Baccalaureate Nursing Students' Perceptions of Inclusive Pedagogical Strategies.” Journal of Professional Nursing 36, no. 4: 229–235. 10.1016/j.profnurs.2019.12.006. 32819549

[nin70054-bib-0032] Morgan, D. L. 2010. “Reconsidering the Role of Interaction in Analyzing and Reporting Focus Groups.” Qualitative Health Research 20, no. 5: 718–722. 10.1177/1049732310364627. 20406996

[nin70054-bib-0033] Moriña, A. 2017. “Inclusive Education in Higher Education: Challenges and Opportunities.” European Journal of Special Needs Education 32, no. 1: 3–17. 10.1080/08856257.2016.1254964.

[nin70054-bib-0034] Motzkau, J. , and E. Schraube . 2015. “Kritische Psychologie: Psychology From the Standpoint of the Subject.” In Handbook of Critical Psychology, edited by Parker and Ian , 280–289. Routledge.

[nin70054-bib-0035] Mørch, L. L. , and L. Huniche . 2006. “Critical Psychology in a Danish Context.” Annual Review of Critical Psychology 5: 192–211. http://www.discourseunit.com/arcp/5.htm.

[nin70054-bib-0036] Nielsen, K. , J. Finderup , L. Brahe , et al. 2017. “The Art of Preceptorship. A Qualitative Study.” Nurse Education in Practice 26: 39–45. 10.1016/j.nepr.2017.06.009. 28668586

[nin70054-bib-0037] Nissen, M. 2000. “Practice Research. Critical Psychology in and Through Practices.” Annual Review of Critical Psychology (Online) 2, no. 2: 145–179. https://discourseunit.com/annual-review/2-2000/.

[nin70054-bib-0038] Owens, R. A. , L. M. Kuhl , C. O. P. Hagopian , et al. 2024. “Professionalism and Professional Identity.” American Nurse Journal 19, no. 2: 14–19. https://www.myamericannurse.com/professionalism-and-professional-identity/.

[nin70054-bib-0039] Patterson, E. E. B. , L. Boyd , and G. Mnatzaganian . 2017. “The Impact of Undergraduate Clinical Teaching Models on the Perceptions of Work‐Readiness Among New Graduate Nurses: A Cross‐Sectional Study.” Nurse Education Today 55: 101–106. 10.1016/j.nedt.2017.05.010. 28575706

[nin70054-bib-0040] Prude, S. B. , R. K. Pecoraro , D. K. Calamia , and E. L. Creel . 2021. “Faculty Attitudes Towards Nursing Students With Disabilities in the Clinical Setting.” Journal of Nursing Education and Practice 11, no. 9: 52. 10.5430/jnep.v11n9p52.

[nin70054-bib-0041] Rodrigues, F. , and M. J. Mogarro . 2019. “Student Teachers' Professional Identity: A Review of Research Contributions.” Educational Research Review 28: 100286. 10.1016/j.edurev.2019.100286.

[nin70054-bib-0042] Sandvik, A. H. , and Y. Hilli . 2023. “Understanding and Formation – A Process of Becoming a Nurse.” Nursing Philosophy 24, no. 1: e12387. 10.1111/nup.12387. 35324066 PMC10078249

[nin70054-bib-0043] Schraube, E. , and C. Højholt . 2016. Psychology and the Conduct of Everyday Life. Routledge. 10.4324/9781315746890.

[nin70054-bib-0044] Schraube, E. , and V. Jartoft . 2017. “Teori gør forskellen: Analytiske strategier i kritisk psykologi.” [Theory Makes the Difference: Analytical Strategies in Critical Psychology] Nordiske Udkast 25, no. 2: 24–39. 10.7146/nu.v25i2.141582.

[nin70054-bib-0045] Skovhus, R. B. , and R. Thomsen . 2022. “Using Critical Psychology in Analysis of Career Guidance and Counselling.” British Journal of Guidance & Counselling 50, no. 4: 491–502. 10.1080/03069885.2022.2050672.

[nin70054-bib-0046] Soerensen, J. , M. Holen , I. S. Jakobsen , P. Larsen , and D. S. Nielsen . 2024. “Safety Means Everything”: An Ethnographic Methodology to Explore the Formation of Professional Identity in Nursing Students.” Nurse Education in Practice 76: 103914. 10.1016/j.nepr.2024.103914. 38364530

[nin70054-bib-0047] Spaan, N. , P. Van Trigt , and A. Schippers . 2024. “Integration of a Disability Lens as Prerequisite for Inclusive Higher Education.” European Journal of Inclusive Education 3, no. 1: 1–24. 10.7146/ejie.v3i1.137271.

[nin70054-bib-0048] Spiers, J. 2000. “New Perspectives on Vulnerability Using Emic and Etic Approaches.” Journal of Advanced Nursing 31, no. 3: 715–721. 10.1046/j.1365-2648.2000.01328.x. 10718892

[nin70054-bib-0049] Suri, H. 2011. “Purposeful Sampling in Qualitative Research Synthesis.” Qualitative Research Journal 11, no. 2: 63–75. 10.3316/QRJ1102063.

[nin70054-bib-0050] Tengelin, E. , E. Dahlborg , I. Berndtsson , and P. H. Bülow . 2020. “From Political Correctness to Reflexivity: A Norm‐Critical Perspective on Nursing Education.” Nursing Inquiry 27, no. 3: e12344. n/a. 10.1111/nin.12344. 32009272

[nin70054-bib-0051] Trede, F. , R. Macklin , and D. Bridges . 2012. “Professional Identity Development: A Review of the Higher Education Literature.” Studies in Higher Education 37, no. 3: 365–384. 10.1080/03075079.2010.521237.

[nin70054-bib-0052] Vabo, G. , Å. Slettebø , and M. Fossum . 2022. “Nursing Students' Professional Identity Development: An Integrative Review.” Nordic Journal of Nursing Research 42, no. 2: 62–75. 10.1177/20571585211029857.

[nin70054-bib-0053] Vestphal, T. K. , K. S. Pedersen , and B. D. Pedersen . 2020. “The Lived Experiences of Emotionally Insecure Nursing Students: A Qualitative Study.” Nurse Education in Practice 43: 102694. 10.1016/j.nepr.2019.102694. 32113178

[nin70054-bib-0054] Warne, T. , U. B. Johansson , E. Papastavrou , et al. 2010. “An Exploration of the Clinical Learning Experience of Nursing Students in Nine European Countries.” Nurse Education Today 30, no. 8: 809–815. 10.1016/j.nedt.2010.03.003. 20409620

[nin70054-bib-0055] World Medical Association . 2025. “World Medical Association Declaration of Helsinki: Ethical Principles for Medical Research Involving Human Participants.” Journal of the American Medical Association 333, no. 1: 71–74. 10.1001/jama.2013.281053. 39425955

