# Supplementary material for: Clinical Supervisors' and Educators' Perspectives on Conditions Forming Nursing Students' Professional Identity: A Qualitative Focus Group Study
Source: Nurs Inq. 2025 Sep 25;32(4):e70054. doi: 10.1111/nin.70054 (PMC12461178; doi:10.1111/nin.70054)
Supplement: Supplementary file 1 — Supplementary information 1. Interview guide. [file NIN-32-e70054-s001.docx]

**Supplementary information 1. Interview guide**

| **1. Understanding conditions and contexts in the formation of professional identity**   - **How do you view the impact of conditions and contexts** in clinical and educational settings on nursing students' ability to develop a professional identity? - **What specific factors** (e.g., institutional resources, workload, social dynamics) influence students’ professional identity formation?   **2. Perceptions of role in the identity formation process**   - **How do you perceive your role** in supporting nursing students’ professional development? - **What conditions or factors** significantly influence your ability to fulfill this role?   - Prompt to discuss the availability of time, organizational support, and institutional culture.   **3. Understanding the identity formation process**   - **In your experience, what process do nursing students undergo** as they develop new identities as professional nurses?   - Follow-up: How do you observe changes in students’ attitudes, behaviors, or perspectives as they progress through this learning trajectory?   **4. Supportive actions for facilitating professional identity**   - **How do you support students in developing their professional identity** despite challenges within clinical and educational environments? |
| --- |
