# Supplementary material for: Clinical Supervisors' and Educators' Perspectives on Conditions Forming Nursing Students' Professional Identity: A Qualitative Focus Group Study
Source: Nurs Inq. 2025 Sep 25;32(4):e70054. doi: 10.1111/nin.70054 (PMC12461178; doi:10.1111/nin.70054)
Supplement: Supplementary file 2 — Supplementary information 2. Illustration of the analysis process. [file NIN-32-e70054-s002.docx]

**Supplementary information 2. Illustration of the analysis process resulting in theme 2,** Perceived student vulnerability and its impact on the learning process

| **Text/data** | **Conditions** | **Meanings** | **Reasons** | **Theme 2** |
| --- | --- | --- | --- | --- |
| "I think our framework is challenged. Also, because the students demand more today. Unfortunately, many students today come with something in their background, whereas before, it was a more homogeneous group. It is not the same today, and that is what we must deal with" [ID 16]. | Diverse and vulnerable student backgrounds; previous homogeneity | The shift to a more diverse student population presents new challenges for participants. | Participants must adapt to support students’ vulnerabilities while maintaining professional standards. | Perceived student vulnerability and its impact on the learning process |
| "I am thinking about the person with care needs, and I had a vulnerable student. Due to GDPR, we received almost no information […] it is important to know certain information to support both the student and protect the patient."[ID18] | The clinical supervisor faces a strict GDPR that limits access to information about the student's needs. This creates a challenging environment for balancing student support and patient safety. | The lack of information is interpreted as a barrier to supporting both the student and the patient, as it hinders the supervisor’s ability to tailor their approach. | The supervisor prioritizes patient safety and adequate support for the students, believing that better information would improve their ability to fulfill these responsibilities. |  |
